# Contrasting soil microbial community functional structures in two major landscapes of the Tibetan alpine meadow

**DOI:** 10.1002/mbo3.190

**Published:** 2014-07-07

**Authors:** Houjuan Chu, Shiping Wang, Haowei Yue, Qiaoyan Lin, Yigang Hu, Xiangzhen Li, Jizhong Zhou, Yunfeng Yang

**Affiliations:** 1 State Key Joint Laboratory of Environment Simulation and Pollution Control, School of Environment, Tsinghua UniversityBeijing, 100084, China; 2 Laboratory of Alpine Ecology and Biodiversity, Institute of Tibetan Plateau Research, Chinese Academy of SciencesBeijing, 100085, China; 3 Key Laboratory of Adaption and Evolution of Plateau Biota, Northwest Institute of Plateau Biology, Chinese Academy of SciencesXining, 810008, China; 4 Shapotou Desert Experiment and Research Station, Cold and Arid Regions and Environmental & Engineering Research Institute, Chinese Academy of SciencesLanzhou, 730000, China; 5 Chengdu Institute of Biology, Chinese Academy of SciencesChengdu, 610041, China; 6 Institute for Environmental Genomics and Department of Botany and Microbiology, University of OklahomaNorman, Oklahoma, 73019; 7 Earth Sciences Division, Lawrence Berkeley National LaboratoryBerkeley, California, 94720

**Keywords:** Alpine grassland, GeoChip, soil microbial community, Tibetan plateau

## Abstract

The grassland and shrubland are two major landscapes of the Tibetan alpine meadow, a region very sensitive to the impact of global warming and anthropogenic perturbation. Herein, we report a study showing that a majority of differences in soil microbial community functional structures, measured by a functional gene array named GeoChip 4.0, in two adjacent shrubland and grassland areas, were explainable by environmental properties, suggesting that the harsh environments in the alpine grassland rendered niche adaptation important. Furthermore, genes involved in labile carbon degradation were more abundant in the shrubland than those of the grassland but genes involved in recalcitrant carbon degradation were less abundant, which was conducive to long-term carbon storage and sequestration in the shrubland despite low soil organic carbon content. In addition, genes of anerobic nitrogen cycling processes such as denitrification and dissimilatory nitrogen reduction were more abundant, shifting soil nitrogen cycling toward ammonium biosynthesis and consequently leading to higher soil ammonium contents. We also noted higher abundances of stress genes responsive to nitrogen limitation and oxygen limitation, which might be attributed to low total nitrogen and higher water contents in the shrubland. Together, these results provide mechanistic knowledge about microbial linkages to soil carbon and nitrogen storage and potential consequences of vegetation shifts in the Tibetan alpine meadow.

## Introduction

Due to its high altitude and extreme climate conditions, the Tibetan plateau is a region vulnerable to the impact of climate changes (Klein et al. 2004; Yu et al. 2006; Qiu 2008; Cui and Graf 2009). The alpine meadow, accounting for roughly 35% of the Tibet plateau, is a representative ecotone of the Tibetan plateau (Cao et al. 2004). The grassland and shrubland are two major landscapes of the alpine meadow. Herbaceous plants such as *Kobresia humilis, Festuca rubra* and *Lancea tibetica* were major species in the grassland, while the shrub species *Potentilla fruticosa* was dominant in the shrubland.

Several studies have been carried out to document vegetation and soil biogeochemical characteristics of these ecotones. The shrubland is typical of higher biomass and thicker canopy than grassland, resulting in higher soil moisture (Du et al. 2008). However, the shrubland was lower in soil organic carbon and methane emission (Jackson et al. 2002; Cao et al. 2008; Yang et al. 2008; Zhang and Gao 2008). The loss of soil organic carbon was substantial enough to offset higher plant biomass carbon, resulting in ecosystem carbon loss when woody plants invaded grasslands (Jackson et al. 2002). For nitrogen, the shrubland was higher in denitrification and N_2_O flux rate but lower in net nitrification rate compared to grassland, suggesting that anerobic processes, in compliance with higher soil moisture, were more substantial in the shrubland (Sun et al. 2005; Du et al. 2008).

Compared to the knowledge of vegetation and soil biogeochemistry at the Tibetan grassland, little knowledge has been obtained for soil microbial community (Chim Chan et al. 2008), despite that soil microbial communities constitute a major biosphere portion of terrestrial ecosystems and play key roles in determining greenhouse gas emission (Falkowski et al. 2008; Singh et al. 2010). Thus, it is necessary to profile soil microbial communities for evaluating the effects and feedback responses of climate or land-use changes. The rapid development of a suite of high-throughput, sequencing- or microarray-based metagenomics tools has enabled accurate measurements of microbial community structures (Call et al. 2003; Curtis and Sloan 2005; Cheung et al. 2006; Claesson et al. 2010). Among these, GeoChip excels in that it efficiently targets a wide range of gene markers involved in carbon, nitrogen, sulfur and phosphorus cycling, antibiotics resistance, metal resistance, and organic pollutant degradation (He et al. 2007; Lu et al. 2012).

In this study, we used GeoChip 4.0, the most advanced version of GeoChip, to profile microbial community structures in the adjacent grassland and shrubland. Specifically, we seek answers to three hypotheses: (1) soil microbial communities are distinct in the grassland and shrubland, given the substantial differences in vegetation and soil biogeochemical properties; (2) environmental properties explain a majority of soil microbial community variations, which might suggest that the harsh environments in the alpine grassland render niche adaptation important; and (3) changes of carbon and nitrogen cycling, and stress genes coincide with soil biogeochemical properties.

## Materials and Methods

### Study site and sampling

The experiment site is located at two adjacent shrubland and grassland areas within Haibei Alpine Meadow Ecosystem Research Station, which is situated on the northeast Qinghai-Tibet Plateau (37°37′N, 101°12′E). Due to the high elevation of 3400 m above sea level, the annual average temperature at this site was −1.7°C, and the annual precipitation was 560 mm (Zhao et al. 2006). Soil pH was 7.3 and 7.4 at the depth of 0–10 cm and 10–20 cm, respectively. Mat Cryic Cambisols was the dominant soil type. Being typical in alpine climate, the vegetation growth season was short, spanning from May to September. The vegetation type is mainly C_3_ species, whose maximal aboveground biomass occurring in late July and early August (Zhao et al. 2006). Among the aboveground vegetation, *P. fruticosa* was the sole shrub species at the study site, with 50–60% total coverage and dominant biomass. The herbaceous species such as *K. humilis, Elymus nulan* and *Festuca ovina* are present in both the grassland and the shrubland with high coverage.

Soil samples were collected in August 2009, with three plots of 2 × 2 m size as replicates for each site. The replicates were roughly 0.6 m from each other in distance. Five soil cores at the depths of 0–10 or 10–20 cm and diameter of 1.5 cm were collected randomly at each plot and mixed well to combine into one composite. Thereafter, soil samples were preserved on ice during transfer to the laboratory, where samples were sieved with 2 mm mesh. A portion of samples was kept at −80°C for GeoChip experiment, and the rest was kept at 4°C or −20°C until environmental property measurements.

### Soil biogeochemical and vegetation measurement

Total soil organic carbon and nitrogen (TOC and TN) at the depths of 0–10 and 10–20 cm were measured by a TOC-5000A analyzer (Shimadzu Corp, Kyoto, Japan) and a Vario EL III Elemental Analyzer (Elementar, Hanau, Germany). The supernatant of soil solution was used for NH_4_^+^-N and NO_3_^−^-N measurement through a FIAstar 5000 Analyzer (FOSS, Hillerd, Denmark) as previously described (Yang et al. 2013).

Aboveground vegetation properties were measured by indices of species number, abundance, diversity, aboveground biomass, and average height based on common protocols (Yang et al. 2013). Among these, the species number and abundance were counted during field sampling. Plant biomass was weighed after mowing. The diversity was calculated by the Shannon–Weaver index (Liu et al. 2014). To measure the average height and coverage of the vegetation canopy, the 1 × 1 m quadrat was divided into 100 0.1 × 0.1 m small squares, then touched species were measured by 0.1 cm marks along a vertical ruler held behind the pin. The canopy height of shrub in 1 × 1 m quadrat was calculated by the average of all species in the zone.

### Soil DNA extraction, purification, and quantitation

FastDNA spin kit for soil (MP Biomedical, Carlsbad, CA) was used to extract soil microbial DNA, following the manufacturer's instructions. Then DNA was precipitated by incubation with 2.5 volumes of 100% ice cold ethanol and 0.1 volume of 3 mol/L NaOAc (pH 5.2) overnight at −20°C and centrifugation for 30 min at 13,000*g*. DNA was dissolved in nuclease-free water. The quality and quantity were measured through a NanoDrop ND-1000 Spectrophotometer (NanoDrop Technologies Inc., Wilmington, DE), PicoGreen (Ahn et al. 1996) and with a FLUOstar Optima (BMG Labtech, Jena, Germany), respectively.

### GeoChip 4.0 hybridization and scanning

DNA samples were labeled with fluorescent dye Cy-5 by a random priming method (Zhao et al. 2014), followedg by purification with a QIA quick purification kit (Qiagen, Valencia, CA). Dye incorporation was measured by a NanoDrop ND-1000 spectrophotometer (NanoDrop Technologies Inc., Wilmington, DE), and DNA was dried by a SpeedVac (ThermoSavant, Milford, MA) at 45°C for 45 min. Thereafter DNA was hybridized with GeoChip 4.0 at 42°C for 16 h in MAUI hybridization station (BioMicro, Salt Lake City, UT) and scanned by a NimbleGen MS200 scanner (Roche, Madison, WI) at 633 nm laser, using 100% and 75% laser power and a photomultiplier tube (PMT) gain, respectively.

### GeoChip data analyses

Data analysis pipeline was used for signal intensity normalization as previously described (He et al. 2010; Yang et al. 2014): (1) to remove poor quality spots flagged as 1 or 3 by ImaGene or with signal to noise ratio (SNR) less than 2.0; (2) to remove genes detected only once among the three replicates; (3) to normalize the data through dividing each spot by total spot intensity of its microarray, then multiply each spot with the average value of microarray's total signal intensity; and (4) then transform the data by natural logarithm.

Principal component analysis (PCA) and the dissimilarity test were performed to examine overall changes in environmental variables, aboveground vegetation, and microbial community structures (Yang et al. 2013). Bray–Curtis distance was used to calculate dissimilarity distance matrices and significance. Mantel test using the Bray–Curtis coefficient and Euclidean distance to construct dissimilarity matrices was performed to examine the relationship between microbial community structure and soil biogeochemical and vegetation properties (Legendre and Legendre 1998). All of the analyses were performed by functions within the vegan package (v.1.17-9) in R (v. 2.12.2) (R Development Core Team. 2011).

## Results

### Distinct plant composition, microbial community and soil properties

The vegetation composition was substantially different at the adjacent shrubland and grassland. Although plant biomass was much higher at the shrubland, its species number and alpha-diversity of the shrubland were significantly lower (*P *<* *0.005) (Table S1). Among the 61 plants species identified at the grassland and shrubland, only 27 species were present at both sites, while 28 and 6 were present only in the grassland and shrubland, respectively. *Potentilla fruticosa* that possessed a predominant biomass of the site was the only shrub species found in the shrubland. For grasses, *K. humilis, L. tibetica, Polygonum viviparum* L., *Potentilla anserina* L. and *Geranium pylzowianum* were major herbaceous species at both sites. The biomass of *K. humilis* and *G. pylzowianum* was higher in the shrubland, while that of *L. tibetica, P. viviparum* L. and *P. anserina* L. was similar for both sites. Regardless, the total biomass of herbaceous species was higher in the grassland as there were substantially more herbaceous species in the grassland.

Distinct vegetation composition between the shrubland and the grassland was verified by the *adonis* dissimilarity test (*P *=* *0.001) (Table1). Furthermore, PCA showed that plants were grouped by the shrubland or grassland and well separated from each other (Fig. S1). Consistently, the beta-diversities between the shrubland and the grassland were substantially higher than those within the shrubland or grassland (Fig. S2).

**Table 1:** The differences of vegetation and microbial community between the shrubland and the grassland based on the dissimilarity test of *adonis*[Table-fn tf1-1].

	Vegetation	Microbial community
Statistic *R*^2^	0.796	0.233
Significance	0.001	0.013

1 *adonis*: nonparametric multivariate analysis of variance (MANOVA) with the *adonis* function. Significance tests were carried out by *F*-tests with sequential sums of squares from permutations of vegetation and GeoChip 4.0 data.

Soil microbial functional structure was examined by GeoChip 4.0. Similar to the observation in plants, substantial difference between the shrubland and the grassland was detected, as shown by the *adonis* dissimilarity test (*P *=* *0.013) (Table1). PCA of GeoChip data indicated that microbial communities of grassland and shrubland were well separated (Fig. S1). Furthermore, beta-diversities of microbial community structures between the shrubland and the grassland were substantially higher than those within the shrubland or grassland (Fig. S2). An average of 37,079 and 27,407 microbial genes were detected in shrubland and grassland, respectively (Table S1). The alpha diversities, indicated by Shannon and Simpson indices, were significantly (*P = *0.001) higher for microbial communities of the shrubland.

As shown in Table S2, TOC, and TN contents at depths of 0–10 cm and 10–20 cm were significantly (*P *=* *0.001) lower in the shrubland, which was consistent with previous observation that the shrubland was nutrient poor (Jackson et al. 2002; Yang et al. 2008; Zhang and Gao 2008). Nevertheless, soil ammonium (NH_4_^+^-N), inorganic nitrogen, and water contents were higher in the shrubland. These differences suggested that soil properties were distinct in both sites.

### The linkage between soil microbial communities and environmental properties

The results of the Mantel test showed that microbial communities were significantly (*P *<* *0.05) correlated to soil and vegetation properties (Table2). Closer examination indicated that microbial communities were significantly (*P *<* *0.05) correlated to soil properties of soil TOC, TN, NH_4_^+^-N, inorganic nitrogen and water contents and C/N ratios, suggesting that carbon and nitrogen inputs play an important role in shaping the microbial community structure (Table S3). In addition, vegetation properties of plant biomass, plant diversity, and species number were correlated with microbial community structure.

**Table 2:** Relationships between microbial community and soil and vegetation properties by partial Mantel tests.

	Soil properties^1^	Vegetation properties^2^
Statistic *r*	0.65	0.48
Significance	0.01	0.04

TOC, total organic carbon; TN, total nitrogen; CN, C/N ratios; SIN, soil inorganic nitrogen.

1 Combination of NO_3_^−^-N10, NH_4_^+^-N10, TOC10, TN10, TOC20, TN20, water20, CN10, CN20, SIN, and N_2_O measurements at the depth of 0–10 cm or 10–20 cm as denoted.

2 Combination of plant biomass, species number and plant diversity.

### Carbon cycling genes

**Figure 1:**
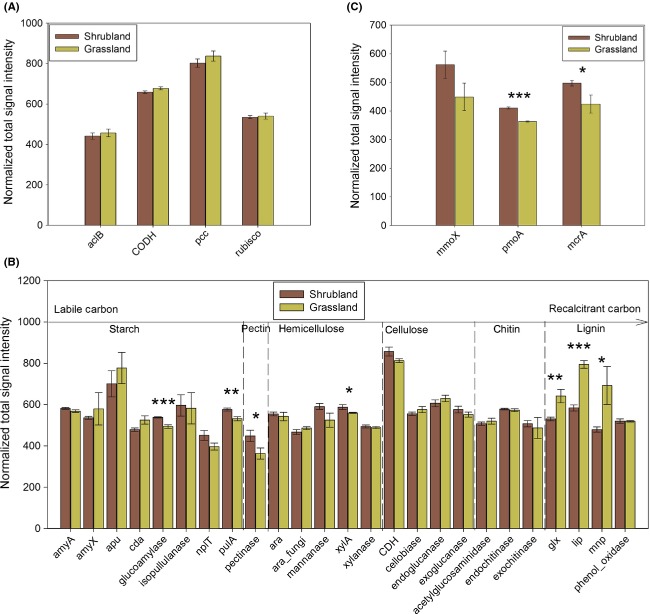
The total abundance of (A) carbon fixation, (B) carbon degradation, and (C) methane cycling genes in the shrubland and grassland. Error bars represented standard error (*n* = 3). The differences between the shrubland and the grassland sites were examined by two-tailed paired *t*-tests. “*” *P *<* *0.10, “**” *P *<* *0.05, “***” *P *<* *0.01. *aclB*, ATP-citrate lyase beta subunit; *CODH*, carbon monoxide dehydrogenase; *pcc*, propionyl-CoA carboxylase; *rubisco*, ribulose-1,5-bisphosphate carboxylase/oxygenase; *mmoX*, methane mono-oxygenase; *pmoA*, particulate methane mono-xygenase alpha subunit; *mcrA*, methyl-coenzyme M reductase alpha subunit; *amyA*, alpha-amylase; *amyX*, pullulanase; *apu*, amylopullulanase; *cda*, cytidine deaminase; *nplT*, neopullulanase; *pulA*, glycogen debranching enzyme; *ara*, alpha-N-arabinofuranosidase; *xylA*, xylose isomerase; *CDH*, cellobiose dehydrogenase; *glx*, glyoxalase; *lip*, triacylglycerol lipase; *mnp*, and manganese peroxidase.

The abundances of carbon fixation genes were largely similar between the shrubland and the grassland (Fig.1A). For carbon degradation genes, total abundances of *glucoamylase* and *pulA* involved in starch degradation, *pectinase* involved in pectin degradation, and *xylA* involved in hemicellulose degradation were significantly (*P*<0.10) higher in the shrubland, but total abundances of lignin degradation genes *glx, lip*, and *mnp* were significantly lower in the shrubland (Fig.1B). The increase of functional potentials of labile carbon degradation and decrease of functional potentials of recalcitrant carbon degradation could lead to low soil organic carbon contents, as verified in Table S2, but are conducive to long-term carbon storage and sequestration in the shrubland.

Total abundances of methane cycling genes *mcrA* and *pmoA* were significantly (*P *<* *0.05) higher in the shrubland (Fig.1C). Out of 131 methane production gene *mcrA* targeted by GeoChip, 50 were detected only in the shrubland soil samples. In contrast, five *mcrA* genes were detected only in the grassland, which indicated a very narrow functional potential for methane production. Almost all of detected *mcrA* genes of the shrubland were derived from uncultured organisms except for *Methanocorpusculum labreanum Z* (Gene ID 124363917) and *Methanoculleus marisnigri JR1* (Gene ID 126178328), which were highly abundant in the shrubland samples. Similarly, most of the methane oxidation genes *mmoX* and *pmoA* were derived from uncultured organisms except for a few (*Methylomicrobium buryatense* (Gene ID 83265614), *Methylocapsa acidiphila* (Gene ID 83308707), *Methylosinus trichosporium* (Gene ID 7188931), *Methylosinus acidophilus* (*Gene ID 67942375*), and *Methylocella silvestris* (Gene ID 217502822)).

### Nitrogen cycling genes

**Figure 2:**
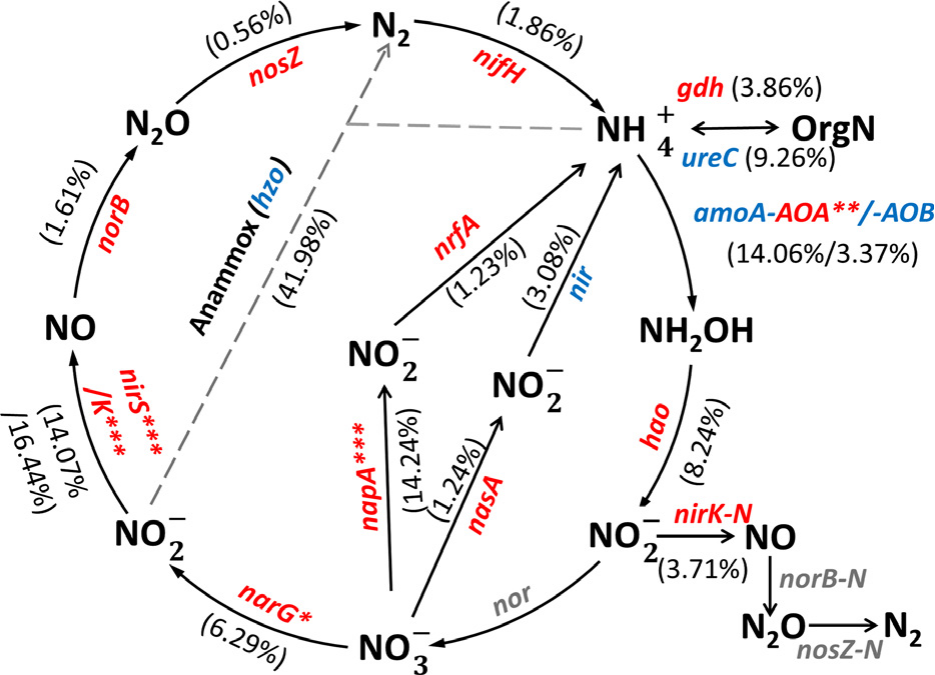
The differences in abundance of N cycling genes in the shrubland and grassland. The percentages in brackets indicate changes in total abundances of functional genes between the shrubland and grassland sites. Red and blue colors represent the higher and lower total abundance when comparing the shrubland samples to the grassland samples, respectively. The gray-colored genes are not targeted by GeoChip 4.0. The differences between the shrubland and grassland sites were examined by two-tailed paired *t*-tests. “*” *P *<* *0.10, “**” *P *<* *0.05, “***” *P *<* *0.01. *nifH*, dinitrogenase reductase; *gdh*, glutamate dehydrogenase; *ureC*, urease alpha subunit; *amoA*, ammonia monooxygenase alpha subunit; *hao*, hydroxylamine oxidoreductase; *nirK*, nitrite reductase; *narG*, nitrate reductase alpha subunit; *nirS*, nitrite reductase; *norB*, nitric oxide reductase; *nosZ*, nitrous-oxide reductase; *hzo*, hydra-zine oxidoreductase; *nasA*, assimilatory nitrate reductase large subunit; *napA*, periplasmic nitrate reductase large subunit; *nrfA*, nitrite reductase.

Abundances of denitrification gene *nirS* and *nirK*, and dissimilatory nitrogen reduction gene *napA* were significantly (*P *<* *0.01) higher in the shrubland, while other nitrogen cycling genes remained similar (Fig.2). Thus, the cycling appeared to shift away from nitrate toward ammonium. Consistently, soil ammonium and inorganic N contents were higher in the shrubland, but soil nitrate content was lower (Table S2).

Abundance of *amoA*-AOA genes, representing the functional potential of ammonia oxidation archaea (AOA), was significantly (*P *<* *0.05) higher by 14.06% on average in the shrubland (Fig.2). In contrast, *amoA*-AOB genes, representing the functional potential of ammonia oxidation bacteria (AOB), remained similar between the shrubland and the grassland. This result suggested that AOA played a more substantial role in nitrification of the shrubland, which could be attributed to low TN content at the site (Table S2) since it has been recently documented that AOA was the major contributor of nitrogen loss under the nitrogen poor condition (Di et al. 2009, 2010). A total of 52 out of 63 *amoA*-AOA genes detected only in the shrubland were derived from *Thaumarchaeota*, a phylum well characterized for its involvement in nitrification (Beman et al. 2008).

### Stress genes

**Figure 3:**
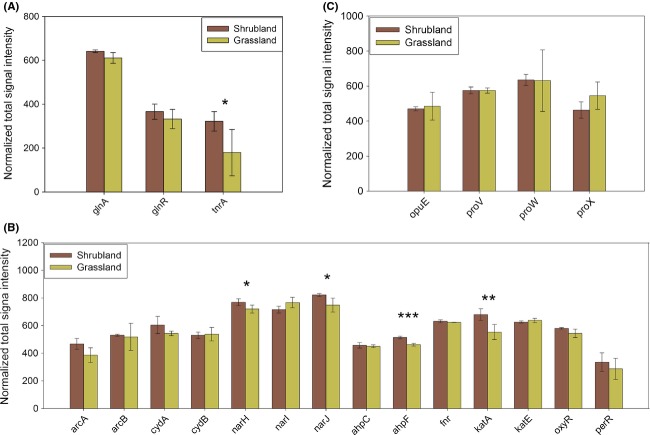
The total abundance of (A) nitrogen limitation, (B) oxygen, (C) osmotic stress genes in the shrubland and the grassland. Error bars represent standard error (*n* = 3). The differences between the shrubland and grassland sites were examined by two-tailed paired *t*-tests. “*” *P *<* *0.10, “**” *P *<* *0.05, “***” *P *<* *0.01. *glnA*, glutamine synthetase; *glnR*, transcriptional repressor of the glutamine synthetase; *tnrA*, nitrogen-sensing transcriptional regulator; *opuE*, osmo-regulated proline transporter; *proV*, glycine betaine transporter subunit; *proW*, glycine betaine transporter subunit; *proX*, glycine betaine transporter subunit; *arcA*, aerobic respiration response regulator of two component signal transduction systems; *arcB*, aerobic respiration response regulator of two component signal transduction systems; *cydA*, cytochrome d terminal oxidase, subunit I; *cydB*, cytochrome d terminal oxidase, subunit II; *narH*, respiratory nitrate reductase beta chain; *narI*, respiratory nitrate reductase gamma chain; *narJ*, respiratory nitrate reductase chaperone NarJ; *ahpC*, alkyl hydroperoxide reductase, C22 subunit; *ahpF*, alkyl hydroperoxide reductase, F52a subunit; *fnr*, oxygen-responsive transcriptional regulator of anerobiosis response; *katA*, component of hydroperoxidase II; *katE*, component of hydroperoxidase II; *oxyR*, transcriptional regulator of LysR family; *perR*, peroxide operon regulator.

The nutrient poor soil in the shrubland could induce microbial responses to nutrient limitation stress, thus we examined the stress genes. The results showed that abundances of all of the three genes responsive to nitrogen limitation (*glnA, glnR* and *tnrA*) were higher in the shrubland, albeit at the significance level of *P *>* *0.05 for *glnA* and *glnR* (Fig.3A). The genes detected only in the shrubland included *glnA* derived from *Roseiflexus* sp. *RS-1* (Gene ID 148656833), *Bacillus* sp. *SG-1* (Gene ID 149181950) and *Roseobacter* sp*. GAI101* (Gene ID 254486307), *glnR* derived from *Alicyclobacillus acidocaldarius LAA1* (Gene ID 218289159), *Staphylococcus carnosus* subsp. *carnosus TM300* (Gene ID 222421052) and *Bacillus amyloliquefaciens FZB42* (Gene ID 154352009), and *tnrA* derived from *B. amyloliquefaciens FZB42* (Gene ID 154351597).

*narH, narJ, ahpF*, and *katA* genes responsive to oxygen limitation were more abundant in the shrubland (Fig.3B), which may be caused by the lower oxygen level due to higher soil water content (Table S2). In contrast, abundances of genes responsive to osmotic stress were largely similar between the shrubland and the grassland (Fig.3C). Thus, osmotic stress was not influential enough to induce microbial responses in the shrubland.

## Discussion

Anthropogenic activities have constantly transformed the Earth's land surface (Vitousek et al. 1997). Among these, a notable consequence is the exchange between woody and herbaceous plants (Jackson et al. 2000). In the alpine meadow of the Tibetan plateau, livestock overgrazing has led to degradation of some shrubland to the grassland (Klein et al. 2007), which in turn alters primary production, nutrient cycling, carbon reallocation, and sequestration (Jobbágy and Jackson 2000). Our GeoChip profiling of microbial community, often considered as the black box for its complexity, filled the knowledge gap. Although GeoChip is subjected to cross-hybridization and unlikely to represent the full breadth of in situ microbial community as it requires preexisting information in the sequence database for probe designing (Yang 2013), it can overcome issues typically associated with sequencing technologies such as random sampling errors (Zhou et al. 2013) and accuracy in quantitative measurements (Tiquia et al. 2004). Therefore, GeoChip has been widely used to dissect functional potentials of microbial communities (Zhou et al. 2011; Yang et al. 2014). In this study, it provides tangible explanations to the differences of soil geochemical properties between the shrubland and the grassland.

The shrubland and the grassland differed substantially in aboveground vegetation, soil water, TOC, TN, and ammonia content (Table S2). In accordance, it was unsurprising to note distinct soil microbial community at these sites (Table1). In general, microbial biomass (García-Orenes et al. 2010), enzyme activity (García-Ruiz et al. 2008), and community diversity (Sharma et al. 2011) were sensitive to changes of environmental conditions and thereby have been tested as indicators of the soil condition (Lagomarsino et al. 2009; Li et al. 2009). These soil microbial parameters have potentials to replace soil geochemical indicators because soil carbon, phosphorus, and potassium varied slowly over time. Here we show that functional composition of microbial communities, as detected by GeoChip, can potentially be used to indicate different environmental conditions as well.

Mantel tests indicated that aboveground vegetation was crucial in shaping microbial community (Table2). As a producer, aboveground vegetation supplies organic resources to soil microbial community, which primarily acts as the decomposer system. Individual vegetation species differs in the quantity and quality of supplied resources and consequently influences the composition of soil microbial community (Hooper et al. 2000; Wardle 2002). Our results of the Mantel tests demonstrated that plant biomass, plant diversity, and species number played important roles in shaping microbial community of the Tibetan alpine meadow (Table S3). Microbial diversity was higher in the shrubland, coinciding with higher plant biomass but opposing to lower plant diversity and species number. Thus, plant biomass might lead to diversification of soil microbial community via reallocation of soil carbon and nitrogen between above and belowground vegetation.

Also, edaphic properties appeared to play a crucial role in shaping microbial community, as indicated by a strong correlation between microbial functional structure and soil organic carbon (*P *<* *0.05) and TN (*P *<* *0.10) contents (Table S3). Nevertheless, correlations between microbial community and edaphic properties should be interpreted with caution due to a complicated interplay between microbial community and soil nutrient cycling (Singh et al. 2010). DNA abundance of functional gene, representing genetic potential, does not necessarily align well with microbial activity and microbe-mediated ecosystem processes because of the interference of DNA remnant from nonviable cells and/or possibly nonlinear scale up from the level of microbial gene or species to the level of ecosystem processes. Bearing in mind these caveats, it was still striking to note the strong correlation between microbial community and soil nutrients, suggesting that soil nutrients were essential in controlling the microbial community structure in the alpine meadow. Notably, relative abundance is used in this study. Differences of gene abundance might be affected by variations of genome size, gene copy number or how well microbial community of the study site is represented by GeoChip probes. Thus, it is unlikely to link gene abundance to the number of microbial species.

Being rich in glaciers and permafrost, Tibet has Earth's third largest ice store that is only after Antarctic and Arctic (Qiu 2010). In accordance, both shrubland and grassland have high soil water content of 56.64 ± 1.56% and 49.45 ± 2.42%, respectively (Table S2). It was shown that soil water content dictated the difference of carbon storage between the shrubland and the grassland (Jackson et al. 2002). The shrubland had a higher TOC in soil than the adjacent grassland at the dry site, and vice versa at the wet site. In accordance, low TOC was detected in the Tibetan shrubland. The low soil organic carbon contents might be caused by the decrease of long-term carbon storage and sequestration in the shrubland as a consequence of shift of microbial functional potential toward labile carbon utilization, as indicated by the increase of genes involved in labile carbon degradation and the decrease of genes involved in recalcitrant carbon degradation (Fig.1B), which was supported by observation that recalcitrant carbon degradation, but not labile carbon degradation, was significantly slower and weaker under low oxygen condition in wet places (Hulthe et al. 1998). Meanwhile, this shift might also be related to the higher soil ammonium content in the shrubland because nitrogen (ammonia and nitrate) input could increase microbial cellulolytic enzyme activity while decreasing microbial ligninolytic enzyme activity during litter decomposition (Carreiro et al. 2000).

For methane emission, microbial genes involved in methane cycling were more abundant in the shrubland. The stimulation of methane cycling could be attributed to higher soil water content, which stimulated anerobic processes. In accordance, an abundance of genes responsive to oxygen limitation was higher (Fig.3B). The net volume of methane emission was contingent on the balance between methane production and consumption. According to a 3-year study It was shown that methane emission at our study sites was significantly lower in the shrubland than in the grassland (Cao et al. 2008).

Higher soil water content also provided an explanation for the stimulation of anerobic nitrogen cycling processes such as denitrification and dissimilatory nitrogen reduction (Peterjohn and Schlesinger 1991), resulting in a shift of soil nitrogen cycling toward ammonium biosynthesis (Fig.2). In accordance, higher denitrification potential was detected in the shrubland compared to the grassland (Zhang et al. 2012). As a consequence, N_2_O emission was shown to be higher in the shrubland (Du et al. 2008), which was also verified by our N_2_O measurements (Table S2).

It was worthy to point out that our study lacked a direct test of vegetation exchange between the shrubland and the grassland independent of other covarying factors. Nonetheless, as the first study to compare functional genes in these two adjacent vegetation landscapes, to our knowledge, it provided valuable clues for predicting consequences of vegetation shifts. A shift between these ecotones, imposed by overgrazing or other anthropogenic/climatic changes, would result in significant changes in microbial community functional potentials and microbe-mediated ecoprocesses such as greenhouse gas emission. Together with changes in soil carbon inputs from aboveground vegetation and oxygen availability, these would inevitably change soil carbon and nitrogen storage in the long term.

## Data Accessibility Section

GeoChip data are available online (http://www.ncbi.nlm.nih.gov/geo/) with the accession number GSE52425.

## Supporting Information

**Figure S1.** PCA (principal component analysis) of vegetation composition and microbial community in the shrubland and the grassland. The values of axis 1 and 2 are percentages of total variations attributed to the corresponding axis. **Figure S2.** Beta-diversity based on comparison on vegetation composition and microbial community in the shrubland and the grassland. The box figures were based on values of Jaccard beta-diversity within the shrubland and the grassland samples, and between the shrubland and the grassland samples. **Table S1.** Diversity indices of vegetation composition and microbial community in the shrubland and the grassland. **Table S2.** Environmental properties in the shrubland and the grassland. **Table S3.** Relationships between microbial community and environmental properties by Mantel tests.
