# Supplementary material for: Contrasting soil microbial community functional structures in two major landscapes of the Tibetan alpine meadow
Source: Microbiologyopen. 2014 Jul 7;3(5):585–94. doi: 10.1002/mbo3.190 (PMC4234253; doi:10.1002/mbo3.190)

**Table S1.** Diversity indices of vegetation composition and microbial community in the shrubland and the grassland.

|  | Indices | Shrubland | Grassland | *P* value |
| --- | --- | --- | --- | --- |
| Vegetation composition | Richness index^a^ | 28.67±0.88 | 39.3±0.88 | **0.001**^d^ |
|  | Shannon Index (H)^b^ | 0.71±0.03 | 3.05±0.09 | **0.001** |
|  | Simpson’ diversity index (1/D) ^c^ | 1.29±0.02 | 16.4±2.31 | **0.003** |
|  | Pielou (Shannon) evenness (J) | 0.21±0.01 | 0.83±0.03 | **0.001** |
|  | Simpson evenness (Si) | 0.04±0.003 | 0.42±0.060 | **0.003** |
| Microbial community | Richness index | 37079±312 | 27407±172 | **0.001** |
|  | Shannon Index (H) | 10.52±0.01 | 10.22±0.01 | **0.001** |
|  | Simpson’ diversity index (1/D) | 36819±294 | 27221±183 | **0.001** |
|  | Pielou (Shannon) evenness (J) | 0.997±0.0001 | 0.997±0.0001 | 1 |
|  | Simpson evenness (Si) | 0.993±0.0003 | 0.993±0.0006 | 0.640 |

^a^Detected plant or gene numbers.

^b^Shannon Index, the higher number, the higher diversity.

^c^Inverse Simpson Index, the higher number, the higher diversity.

^d^Value of significance. Values of *P*<0.05 are marked in bold.

**Table S2.** Environmental properties in the shrubland and the grassland.

| Environmental property | Shrubland | Grassland | *P* value |
| --- | --- | --- | --- |
| NO_3_^-^-N10^a^ (mg·kg^-1^) | 0.850±0.161 | 0.668±0.122 | 0.410^b^ |
| NH_4_^+^-N10 (mg·kg^-1^) | 4.079±0.117 | 2.898±0.146 | **0.003** |
| TOC10 (g·kg^-1^) | 62.40±0.24 | 73.10±0.32 | **0.001** |
| TN10 (g·kg^-1^) | 5.19±0.03 | 5.70±0.05 | **0.001** |
| TOC20 (g·kg^-1^) | 30.70±0.29 | 52.20±0.35 | **0.001** |
| TN20 (g·kg^-1^) | 3.20±0.01 | 3.74±0.01 | **0.001** |
| Water20 % | 56.64±1.56 | 49.45±2.42 | 0.067 |
| CN10 | 12.02±0.10 | 12.82±0.09 | **0.004** |
| CN20 | 9.61±0.06 | 13.96±0.08 | **0.001** |
| SIN10 (mg·kg^-1^) | 4.929±0.115 | 3.566±0.200 | **0.004** |
| N_2_O (ug.m^-2^.h^-1^) | 3.853±1.874 | 1.542±2.149 | 0.460 |
| Biomass (g) | 285.84±8.34 | 34.05±3.23 | **0.001** |
| Species | 20.3±0.7 | 25.9±0.8 | **0.006** |
| Diversity | 0.71±0.02 | 3.05±0.08 | **0.001** |

^a^Abbreviation: NO_3_^-^-N10 and NH_4_^+^-N10 represented nitrate and ammonium at the depth of 0-10 cm, TOC10, TN10, TOC20 and TN20 represented TOC (Total organic C) and TN (Total N) at the depths of 0-10 cm and 10-20 cm, respectively, CN10 and CN20 represented TOC/TN ratio at the depths of 0-10 cm and 10-20 cm, respectively, Water20 represented soil water content at the depth of 10-20 cm, SIN10 (Soil Inorganic Nitrogen) is the sum of NO_3_^-^-N10 and NH_4_^+^-N10, N_2_O represented N_2_O flux measured during sampling, Biomass, Species and Diversity are measured as plant dry weight, species numbers and Shannon diversity of the vegetation respectively.

^b^Value of significance. Values of *P*<0.05 are marked in bold.

**Table S3.** Relationships between microbial community and environmental properties by Mantel tests.

| **Soil property** | NO_3_^-^-N10^a^ | NH_4_^+^-N10 | TOC10 | TN10 | TOC20 | TN20 |
| --- | --- | --- | --- | --- | --- | --- |
|  | -0.046 | 0.891**^b^ | 0.990** | 0.973*** | 0.994** | 0.989* |
|  | water20 | CN10 | CN20 | SIN | N_2_O |  |
|  | 0.421*** | 0.846* | 0.995*** | 0.878** | -0.162 |  |
| **Vegetation property** | Biomass | Species | Diversity |  |  |  |
|  | 0.985* | 0.837* | 0.98** |  |  |  |

^a^Abbreviation: NO_3_^-^-N10 and NH_4_^+^-N10 represented nitrate and ammonium at the depth of 0-10 cm, TOC10, TN10, TOC20 and TN20 represented TOC (Total organic C) and TN (Total N) at the depths of 0-10 cm and 10-20 cm, respectively, CN10 and CN20 represented TOC/TN ratio at the depths of 0-10 cm and 10-20 cm, respectively, Water20 represented soil water content at the depth of 10-20 cm, SIN10 (Soil Inorganic Nitrogen) is the sum of NO_3_^-^-N10 and NH_4_^+^-N10, N_2_O represented N_2_O flux measured during sampling, Biomass, Species and Diversity are measured as plant dry weight, species numbers and Shannon diversity of the vegetation respectively.

^b^“*” *P*<0.10，“**” *P*<0.05，“***” *P*<0.01.

**Figure S1.** PCA (Principal Component Analysis) of vegetation composition and microbial community in the shrubland and the grassland. The values of Axis 1 and 2 are percentages of total variations attributed to the corresponding axis.


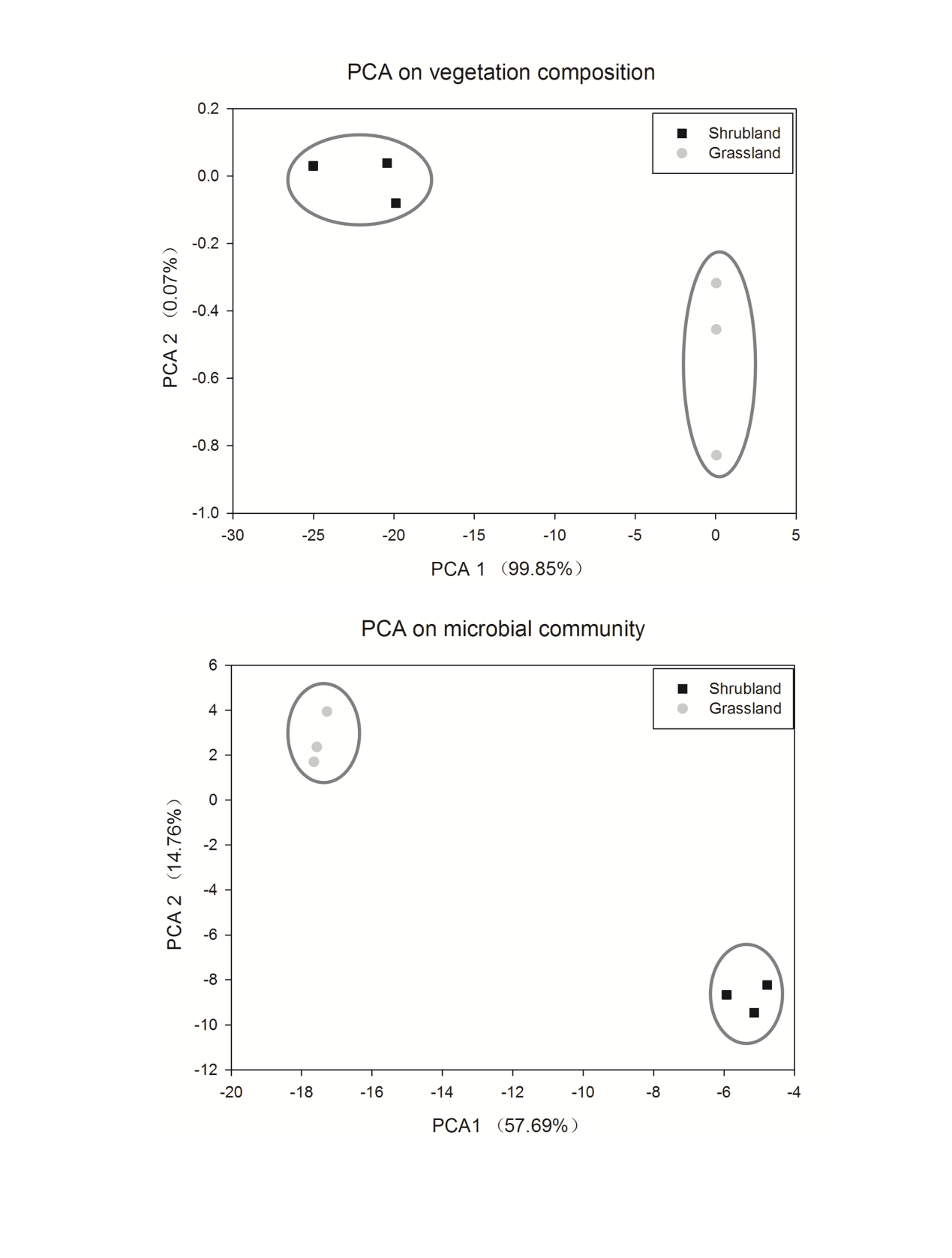


**Figure S2. Beta-diversity based on comparison on vegetation composition and microbial community in the shrubland and the grassland.** The box figures were based on values of Jaccard beta-diversity within the shrubland and the grassland samples, and between the shrubland and the grassland samples.


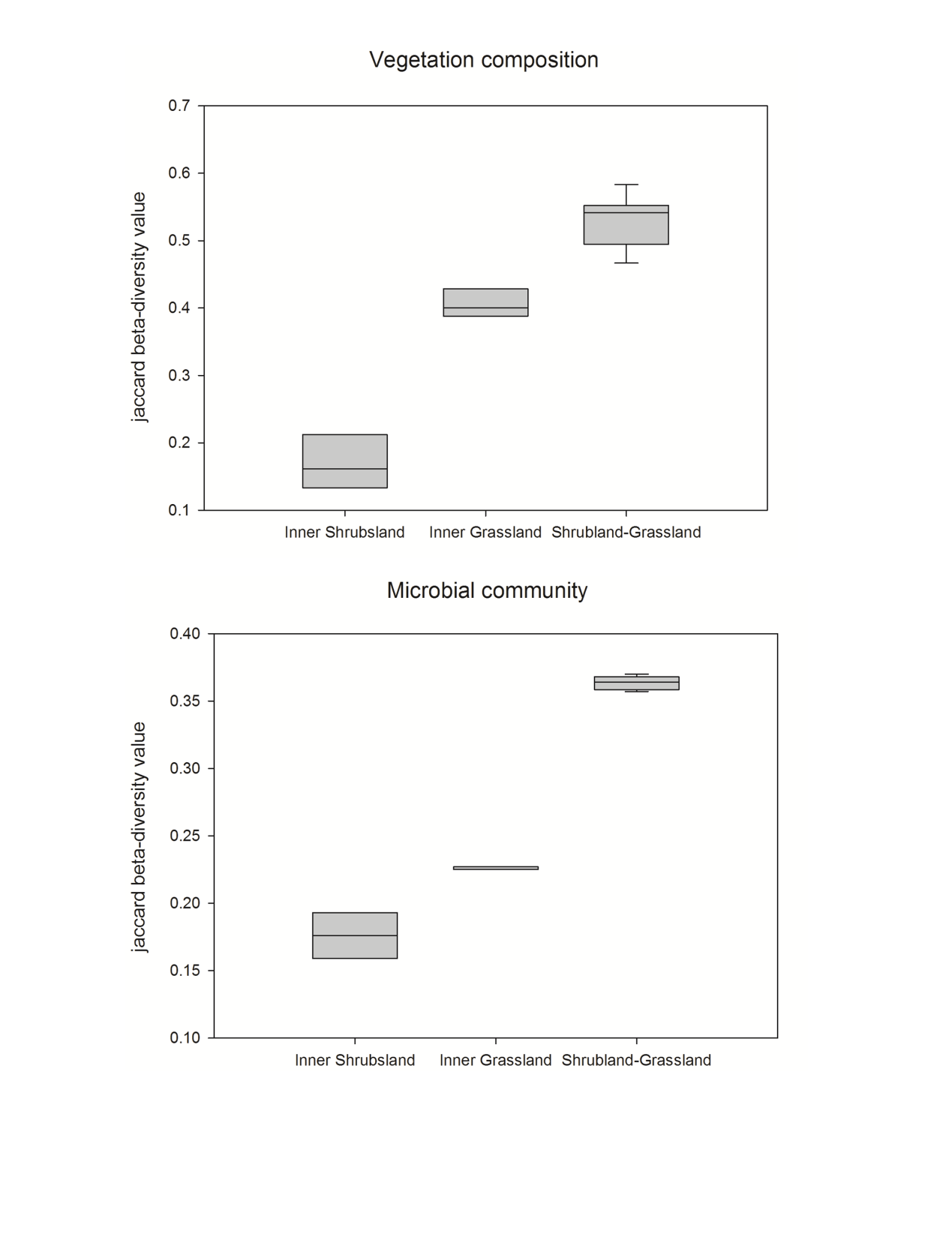

Supplement: Supplementary file 1 — Figure S1. PCA (principal component analysis) of vegetation composition and microbial community in the shrubland and the grassland. The values of axis 1 and 2 are percentages of total variations attributed to the corresponding axis. Figure S2. Beta-diversity based on comparison on vegetation composition and microbial community in the shrubland and the grassland. The box figures were based on values of Jaccard beta-diversity within the shrubland and the grassland samples, and between the shrubland and the grassland samples. Table S1. Diversity indices of vegetation composition and microbial community in the shrubland and the grassland. Table S2. Environmental properties in the shrubland and the grassland. Table S3. Relationships between microbial community and environmental properties by Mantel tests. [file mbo30003-0585-sd1.docx]
